# The biological context of HIV-1 host interactions reveals subtle insights into a system hijack

**DOI:** 10.1186/1752-0509-4-80

**Published:** 2010-06-07

**Authors:** Jonathan E Dickerson, John W Pinney, David L Robertson

**Affiliations:** 1 Faculty of Life Sciences, University of Manchester, Oxford Road, Manchester, M13 9PT, UK; 2 Division of Molecular Biosciences, Imperial College London, South Kensington Campus, London SW7 2AZ, UK

## Abstract

**Background:**

In order to replicate, HIV, like all viruses, needs to invade a host cell and hijack it for its own use, a process that involves multiple protein interactions between virus and host. The HIV-1, Human Protein Interaction Database available at NCBI's website captures this information from the primary literature, containing over 2,500 unique interactions. We investigate the general properties and biological context of these interactions and, thus, explore the molecular specificity of the HIV-host perturbation. In particular, we investigate (i) whether HIV preferentially interacts with highly connected and 'central' proteins, (ii) known phenotypic properties of host proteins inferred from essentiality and disease-association data, and (iii) biological context (molecular function, processes and location) of the host proteins to identify attributes most strongly associated with specific HIV interactions.

**Results:**

After correcting for ascertainment bias in the literature, we demonstrate a significantly greater propensity for HIV to interact with highly connected and central host proteins. Unexpectedly, we find there are no associations between HIV interaction and inferred essentiality. Similarly, we find a tendency for HIV not to interact with proteins encoded by genes associated with disease. Crucially, we find that functional categories over-represented in HIV-host interactions are innately enriched for highly connected and central proteins in the host system.

**Conclusions:**

Our results imply that HIV's propensity to interact with highly connected and central proteins is a consequence of interactions with particular cellular functions, rather than being a direct effect of network topological properties. The lack of a propensity for interactions with phenotypically essential proteins suggests a selective pressure to minimise virulence in retroviral evolution. Thus, the specificity of HIV-host interactions is complex, and only superficially explained by network properties.

## Background

Human immunodeficiency virus type 1 (HIV-1) and its associated illnesses have major health and socio-economic impacts, particularly in developing countries [1]. Concomitant with the progression of the HIV pandemic there has, thus, been a major international research effort, leading to a detailed understanding of HIV biology. One of the most important aspects of this knowledge is the set of known contacts between viral proteins and the host system[2-4], fundamental to HIV's life cycle. HIV, like all viruses, subjugates and exploits host cells in order to propagate. To achieve this, the HIV virion must first bind to a host cell, primarily CD4+ T cells, macrophages and dendritic cells, and then 'hijack' their cellular machinery [5]. Untreated HIV infection leads to a decrease in CD4+ T cell count, eventually resulting in the loss of cell-mediated immunity, an immunocompromised state and the onset of AIDS (Acquired Immunodeficiency Syndrome) [6]. However, infection with the HIV-like simian immunodeficiency virus (SIV) in its "natural" hosts, does not generally result in the development of AIDS, even when viral loads are high [7]. Despite SIV exhibiting high viral loads, and there being a decreased CD4+ T cell count in natural hosts, these infections are effectively non-pathogenic. The differences between natural and human hosts must, thus, be due to the molecular specificity of viral perturbation of the host system: that is the gain (or loss) of protein-protein interactions during adaptation to different host species or because these host systems differ themselves.

More general work on the use of the host system by pathogens [8] has found patterns in the types of interactions and infection strategies employed by multiple pathogens. Specifically, pathogens appear to preferentially interact with "key" human proteins that already participate in multiple interactions and/or have central importance in intra-cellular communication. Highly connected proteins, or "hubs", have classically characterised vulnerable points in a network due to their role in a large number of interactions and due to their association with essentiality [9-11]. Similarly, "bottlenecks", that is proteins with a high betweenness centrality, a measure of the total number of shortest paths going through the protein [12,13], also associate with protein essentiality [14-16]. It has been inferred that this non-uniform contact with the host system represents evolutionary pressure to optimise exploitation of the host cell [8].

In order to test the hypothesis that the specificity of HIV interactions is in some way explained by network properties, we examine their biological context by integrating known phenotypic properties. Our analysis is based on the HIV-1, Human Protein Interaction Database (HHPID), which currently comprises over 2,500 unique interactions, curated from over 3,200 papers with over half of the interactions validated by being linked to multiple publications [2,4]. While this data set no doubt contains false positive interactions and potential bias, it nevertheless constitutes an excellent catalogue of HIV-human interactions as represented by published research [3].

In terms of phenotypic properties, whilst it is difficult to assess human gene essentiality directly, we can use mouse genome knockout data as a proxy for the importance of a gene in terms of a known phenotypic consequence in disrupting its product's function [17]. Similarly, gene-disease associations from The Online Mendelian Inheritance in Man (OMIM)[18] provide another cohort of genes for which deleterious mutations are associated with phenotypic consequence. Integrating these phenotypic data into our network would be expected to corroborate any relationships with topological properties, since proteins with a high connectivity and high betweenness centrality have been demonstrated as having a tendency to be essential [9-11,14-16].

Correcting for ascertainment bias, however, we find that there is no significant relationship between HIV interaction and protein essentiality, and there is a potential under-representation of disease-association amongst HIV interacting human proteins. Moreover we find that HIV's propensity to interact with highly connected and central proteins is most probably a consequence of interactions with specific cellular functions. Thus, the biological context of HIV-interacting proteins, rather than their individual properties, has been the key determinant in the infection of hosts by retroviruses.

## Results

### HIV tends to interact with key host proteins such as bottlenecks and hubs

**Figure 1:**
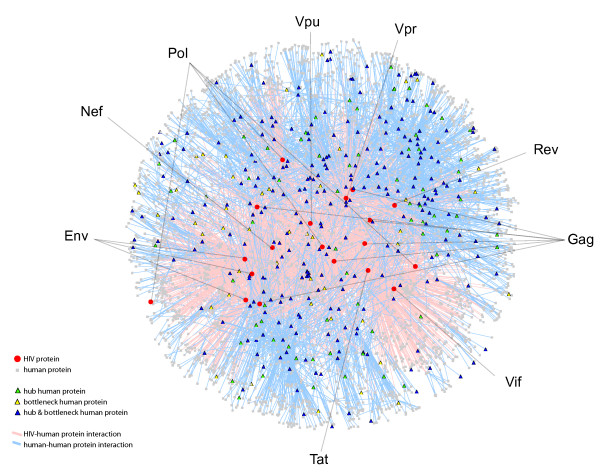
**Subset of the HIV-host interaction network**. Red circles and grey squares correspond to HIV-1 (n = 19) and human proteins, respectively (n = 3,118). Human proteins that are hubs (degree ≥23) or bottlenecks (betweenness ≥2.43 × 10^-04^) are shown as green triangles (n = 69) and yellow triangles (n = 45), respectively. Human proteins that are hubs and bottlenecks are shown as blue triangles (n = 261). Pink edges correspond to interactions between HIV-1 and human proteins (n = 2588) whilst blue edges correspond to human-human interactions (n = 3800), where one of the proteins interacts with HIV-1. HIV-1 proteins are labelled.

**Figure 2:**
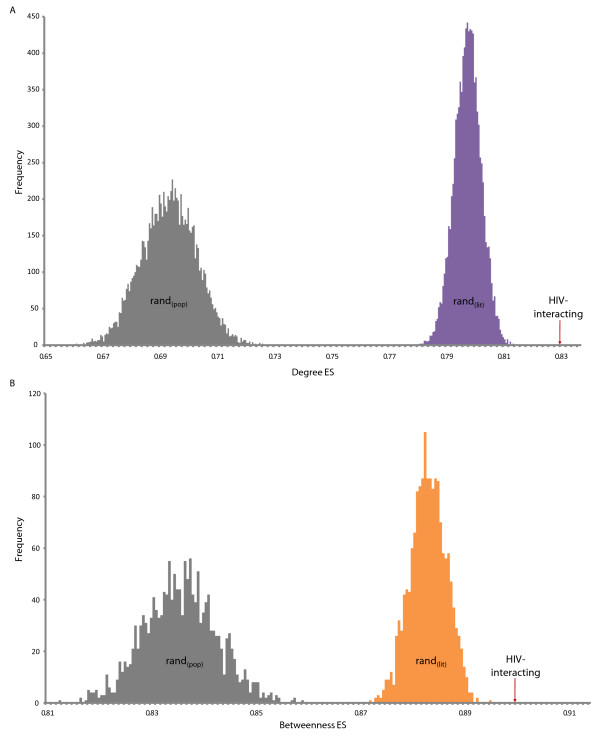
**A) Degree enrichment amongst HIV and randomised data sets**. Distributions of enrichment scores (ES), from gene set enrichment analysis (GSEA), for the degree of 10,000 random samples taken from the population of protein-coding genes, *rand*_*(pop) *_(grey) and 10,000 randomised samples taken to match the publication count distribution of the HIV proteins *rand*_*(lit) *_(purple). The average ES amongst the *rand*_*(pop) *_sample is 0.69 (p-value of 8.90 × 10^-48^) whilst that of *rand*_*(lit) *_is 0.80 (p-value of 6.63 × 10^-15^). **B) Betweenness enrichment amongst HIV and randomised data sets**. Distributions of ES for the betweenness centrality of *rand*_*(pop) *_(grey) and *rand*_*(lit) *_(orange). The ES_(betweenness) _of the HIV-interacting proteins (red arrow) is 0.90 and the average amongst the *rand*_*(pop) *_sample is 0.84 (p-value of 1.98 × 10^-21^) whilst that of *rand*_*(lit) *_is 0.88 (p-value of 4.36 × 10^-8^).

The HIV-interacting human proteins are known to interact with approximately 6,000 other human proteins when integrated with the composite human protein interaction data set from NCBI, dramatically highlighting the highly connected nature of the HIV-host interactions and their neighbours [4] (Figure 1). Proteins with a high degree (connectivity) are involved in a large number of interactions and have been previously shown to be associated with essentiality [9-11]. We used the Gene Set Enrichment Analysis (GSEA) algorithm [19] to determine if the degree distribution of HIV-interacting proteins, taken from HHPID [4], is greater than for random sets of genes taken from the human proteome. We calculate the enrichment scores (ES, from GSEA) for the degree of HIV-interacting proteins, in addition to 10,000 random samples (each of the same size, see methods) taken from the protein-coding gene population *(rand*_*(pop)*_). The ES_(degree) _for HIV-interacting proteins was 0.83, significantly higher than the average amongst the *rand*_*(pop) *_sample, 0.69 (p-value of 8.90 × 10^-48^) (Figure 2A, grey distribution). Note, a higher ES denotes a stronger tendency towards higher degrees. This result confirms a previously reported propensity for HIV to interact with highly connected proteins [8].

**Figure 3:**
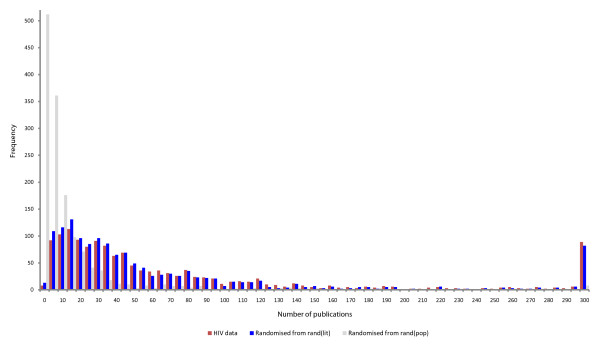
**Rejection sampling versus random sampling**. Average publication distributions for 10,000 random samples taken from the population of protein-coding genes, *rand*_*(pop) *_(grey) and 10,000 randomised samples taken to match the publication count distribution of the HIV sample, *rand*_*(lit) *_(blue). The *rand*_*(lit) *_samples match the HIV publication distribution with a p-value of 0.43 (chi-squared).

However, there will potentially be substantial bias in these results due to the nature of literature curation [20]. In investigations such as this, we are reliant on accurate data to annotate our interactions. It is feasible that these genes, and their annotations, are influenced by ascertainment bias in the literature. Specifically, highly studied genes and proteins, particularly those associated with medically important molecules, can bias results. To compensate for this, we devised a novel method to evaluate ascertainment bias based on a particular gene's publication count in PubMed. If a gene has a high publication count, it is inferred that it is highly studied, and there is therefore a greater chance of observing an interaction. Randomly sampling from a population without correcting for publication counts does not offer fair comparison between the control and (biased) query samples (Figure 3). That is, HIV-interacting proteins are more likely to be highly studied and hence have had greater secondary analysis leading to greater annotation. The control set should be equally studied to offer a fair comparison. Accordingly, we calculated the ES for the degree of HIV-interacting proteins amongst 10,000 randomised samples taken to match the publication count distribution of the HIV sample (*rand*_*(lit)*_). Figure 2A (purple distribution) shows these distributions. The ES_(degree) _of the HIV-interacting proteins is 0.83 and the average amongst the *rand*_*(pop) *_sample is 0.69 (p-value of 8.90 × 10^-48^) whilst that of *rand*_*(lit) *_is 0.80 (p-value 6.63 × 10^-15^). Thus, even after correcting for ascertainment bias, we can confirm that HIV tends to interact with proteins that have a high degree.

Numerous studies have suggested that betweenness centrality also has some significance for the properties of proteins [14-16]. Does HIV preferentially interact with proteins that have a high betweenness? We calculated the ES of the betweenness centrality (in the same way as for degree), amongst the sample data sets. The ES_(betweenness) _of HIV-interacting proteins is 0.90 and the average ES amongst the *rand*_*(pop) *_sample is 0.84 (p-value of 1.98 × 10^-21^), whilst that of *rand*_*(lit) *_is 0.88 (p-value of 4.36 × 10^-8^). Again, despite a significant difference between *rand*_*(pop) *_and *rand*_*(lit)*_, HIV-interacting proteins can be shown to have a higher betweenness centrality than expected (Figure 2B).

To highlight the consequence of the betweenness centrality/degree overlap, a partial human-human protein interaction network visualisation was created using HIV-host interactions from HHPID (pink edges) and then incorporating any additional human-human interactions the human partner has (blue edges) from NCBI (see methods). This was annotated with nodes that are hubs (high degree), bottlenecks (high betweenness centrality) or both hubs and bottlenecks (Figure 1). Furthermore, HIV-interacting over-representation was demonstrated in the full network (n = 21,504) amongst hubs but not bottlenecks (n = 92) and conversely bottlenecks but not hubs (n = 85) and was found to be 51.09% (p-value of 1.34 × 10^-28^) and 32.94% (p-value of 6.72 × 10^-12^) respectively.

These results raise some questions: why has HIV evolved to preferentially interact with key host proteins? Is HIV preferentially interacting with functionally "essential" proteins, as has been suggested for pathogens generally [8]?

### HIV-interacting genes have no relationship with essentiality

**Figure 4:**
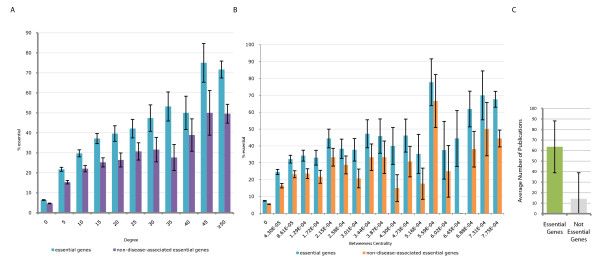
**Essentiality and topological properties of the human network**. (A) Relationship between protein degree and essentiality. Protein degree and the percentage of essential genes (blue) demonstrate a positive linear relationship (p-value of 2.52 × 10^-6^, r^2 ^= 0.92). Excluding overlapping disease-associated genes from the essential set (purple) does not alter the relationship (p-value of 9.87 × 10^-6 ^r^2 ^= 0.89). (B) Relationship between protein betweenness centrality and essentiality. As for panel A, but for betweenness centrality. The percentage of essential genes (blue) demonstrate a positive linear relationship with betweenness (p-value of 5.86 × 10^-5^, r^2 ^= 0.90). Similarly excluding overlapping disease-associated genes from the essential set (orange) does not alter the relationship, (p-value of 6.14 × 10^-5^, r^2 ^= 0.88). (C) Relationship between publication count and essentiality. Essential genes (green) have an average greater publication count than non-essential genes (grey): 64 to 14 publications, respectively.

To test this premise for the human interaction network, we explored the relationship between protein essentiality, degree and betweenness centrality, using mouse genome knockout data as a suitable proxy for essentiality [17]. We assume that a human gene can be considered essential if a knockout of its mouse ortholog confers lethality [21]. We find that there is a positive correlation between protein connectivity and essentiality (Figure 4A, p-value of 2.52 × 10^-6^, r^2 ^= 0.92) in the human protein interaction network. Similarly, a positive correlation exists between protein betweenness centrality and essentiality (Figure 4B, p-value of 5.86 × 10^-5^, r^2 ^= 0.90). These relationships are unaffected by a 28% (n = 603) overlap with disease-associated genes as their exclusion from the data did not alter the displayed trend (Figure 4A, p-value of 9.87 × 10^-6^, r^2 ^= 0.89 and 3B, p-value of 6.14 × 10^-5^, r^2 ^= 0.88). This suggests that the greater the protein degree or betweenness centrality, the greater the likelihood of essentiality, as previously characterised [9-11,14-16].

**Figure 5:**
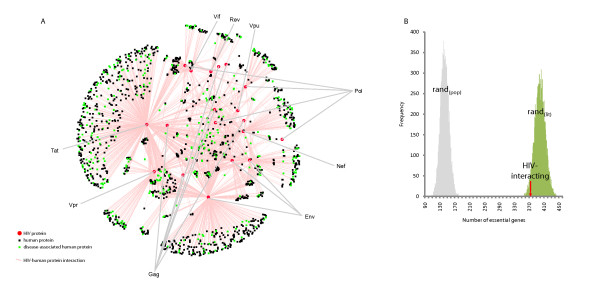
**Essentiality and HIV-interacting proteins**. (A) Visualisation of essential proteins amongst HIV-interacting proteins. Green squares correspond to human proteins identified as essential from mouse knockout data. Black squares and red circles correspond to human and HIV proteins, respectively. Pink edges correspond to interactions between HIV-1 and human proteins, as shown in Figure 4. HIV proteins are labelled accordingly. (B) Number of essential proteins amongst HIV and randomised data sets. Without correcting for bias, *rand*_*(pop)*_contained an average 143 (9.99%) essential proteins, compared to 376 (26.28%) essential proteins in the HIV set (p-value of 3.86 × 10^-95^). When the bias is corrected for, *rand*_*(lit)*_contains an average 399 (27.88%) essential genes, whilst being under-represented, the result is not significant (p-value of 0.0574) and is hence similar to the HIV-1 interacting sample.

We have confirmed that proteins with a high degree are more likely to be HIV-interacting and more likely to be essential (Figure 2 and 4). We might therefore expect to see an over-representation of essentiality amongst HIV-interacting proteins. Integrating homolog mouse genome knockout data [17] with the HIV interactions reveals that HIV interacts with 376 (26.28%) essential proteins (Figure 5A). Furthermore, the mean number of essential proteins seen in *rand*_*(pop) *_is only 143 (9.99%) (Figure 5B). There is therefore an apparent overrepresentation of essentiality amongst the HIV-interacting genes (p-value of 3.86 × 10^-95^). Investigating the PubMed statistics for essential and non-essential genes reveals that essential genes have on average a far greater publication count (mean 64 to 14, respectively; Figure 4C). Thus, inferences about the relationships between essentiality and degree are potentially distorted. Correcting for ascertainment bias using the method previously described, the mean number of essential genes in *rand*_*(lit) *_is 399 (27.88%), similar to the number amongst HIV-1 interacting proteins. Whilst being under-represented, the result is not significant (p-value of 0.0574). This illustrates how failure to compensate for ascertainment bias may affect the overall result. Unexpectedly, our results therefore do not support any significant relationship between HIV-1 interaction and essentiality: HIV appears to be no more or less likely to interact with essential genes than other genes, despite their high connectivity and high betweenness centrality.

### HIV-interacting genes tend not to be disease-associated

**Figure 6:**
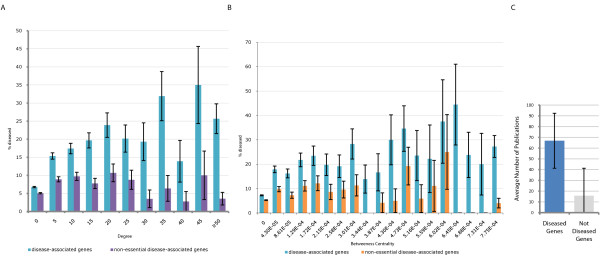
**Disease association and topological properties of the human network**. (A) Relationship between protein degree and disease-association. Protein degree and the percentage of disease-associated genes (blue) demonstrate a slight positive linear relationship (p-value of 0.02). Excluding overlapping essential genes from the disease-associated set (purple) removes the relationship (p-value of 0.29). (B) Relationship between protein betweenness centrality and disease-association. As for panel A, but for betweenness centrality. The percentage of disease-associated genes (blue) demonstrate a positive linear relationship with betweenness, (p-value of 0.01). Similarly excluding overlapping essential genes from the disease-associated set (orange) removes the relationships, (p-value of 0.45). (C) Relationship between publication count and disease-association. Disease-associated genes (green) have an average greater publication count than non-disease-associated genes (grey), 67 to 16 publications, respectively.

Disease associated genes have previously been shown to display a limited propensity towards encoding hub proteins [22]. To test this, we explored the relationship between disease-association and connectivity (Figure 6A), in addition to betweenness centrality (Figure 6B). We find that, whilst there is a slight positive correlation between degree and disease-association (Figure 6A, p-value of 0.02, r^2 ^= 0.47), this disappears when the 33% (n = 603) overlap with essentiality is removed (Figure 6A, p-value of 0.29, r^2 ^= 0.13). Disease-association and betweenness centrality (Figure 6B, p-value of 0.01, r^2 ^= 0.33) are also less correlated when the overlap is removed (Figure 6B, p-value of 0.45, r^2 ^= 0.04).

**Figure 7:**
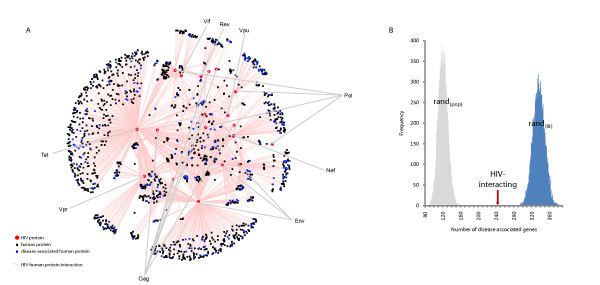
**Disease association and HIV-interacting proteins**. (A) Visualisation of disease-associated genes amongst HIV-interacting proteins. Blue squares correspond to human proteins identified in OMIM as being disease-associated. Black squares and red circles correspond to human and HIV proteins, respectively. Pink edges correspond to interactions between HIV-1 and human proteins, as shown in Figure 4. HIV proteins are labelled accordingly. (B) Number of disease associated genes amongst HIV and randomised data sets. Without correcting for bias, *rand*_*(pop)*_contained an average 120 (8.39%) disease-associated genes, compared to 244 (17.05%) disease-associated genes in the HIV set (p-value of 8.58 × 10^-32^). When the bias is corrected for, *rand*_*(lit)*_contains an average 336 (23.48%) disease-associated genes, which is different to the HIV-1 interacting sample (p-value of 3.48 × 10^-12^).

Without correcting for bias, it appears that gene disease association obtained from OMIM [18] is significantly over-represented amongst HIV-interacting proteins when compared to non-HIV-interacting proteins (Figure 7). Of the HIV-1 interacting human genes, 244 (17.05%) are associated with disease, whilst 120 (8.39%) of the randomised samples (*rand*_*(pop)*_) on average are disease-associated (p-value of 8.58 × 10^-32^). Investigating the publication count in PubMed for disease-associated and non-disease-associated genes reveals that disease-associated genes generally have larger numbers of publications (mean 67 versus 16; Figure 6C), again indicating potential bias. Correcting for this, the literature-count matched random set (*rand*_*(lit)*_) has a mean of 336 (23.48%) disease-associated genes (p-value of 3.48 × 10^-12^). Contrary to the initial result, this suggests that there may in fact be fewer interactions with disease-associated genes than expected (Figure 7B).

### HIV-interacting proteins are over-represented for fundamental biological processes

To place our findings in a stronger biological context, we next investigated the relationship between HIV-host interactions and protein function. A functional understanding of the host-pathogen interaction network can be gained by integrating annotations from GO [4,23]. To investigate HIV's use of the host system in more detail, we identified biological processes over-represented for HIV interactions (see also Pinney *et al*. [3]). These categories represent diverse functions exploited by multiple interactions, involving multiple HIV genes, demonstrating that HIV proteins co-ordinate to target specific parts of the human cellular system.

**Figure 8:**
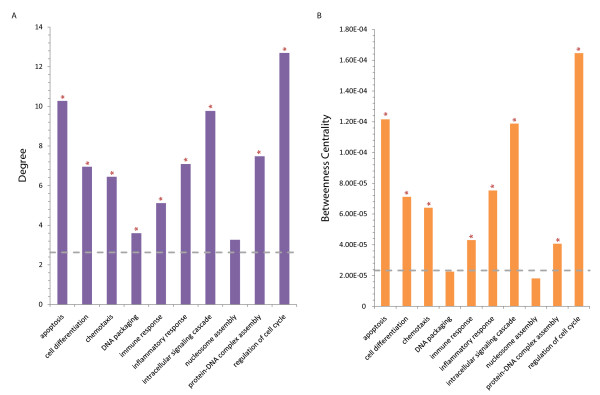
**Protein degree (A) and betweenness centrality (B) for proteins involved in key cellular processes**. The degree and betweenness centrality for proteins involved in the key over-represented biological processes GO terms from [4]. Grey dashed lines correspond to the average degree (2.63) and betweenness centrality (2.33 × 10^-5^) amongst the human-human protein interaction network. The functionally over-represented proteins have a mean degree and betweenness of 7.27 and 7.40 × 10^-5^, respectively. P-values (from Wilcoxon rank-sum test) < 0.05 are indicated by an asterisk (*) above the data-points, suggesting the degree/betweenness distribution for each GO term is significantly different than that for all GO terms.

To investigate HIV's propensity to interact with key proteins, we determined the degree and betweenness centrality for proteins involved in the over-represented biological process GO terms, including immune and apoptotic processes (Figure 8). We find proteins with these terms are generally more highly connected and central. The mean degree and betweenness centrality amongst the human protein interaction network was found to be 2.63 and 2.33 × 10^-5^, respectively, whilst those of the functionally over-represented proteins are 7.27 and 7.40 × 10^-5^, respectively. This finding that human proteins within the functional classes that HIV interacts with most often are themselves more likely to be hubs and bottlenecks accounts for the observed tendency of HIV-interacting proteins to be highly connected and central and is independent of the genes' essentiality or disease association.

### HIV's use of the host system

**Figure 9:**
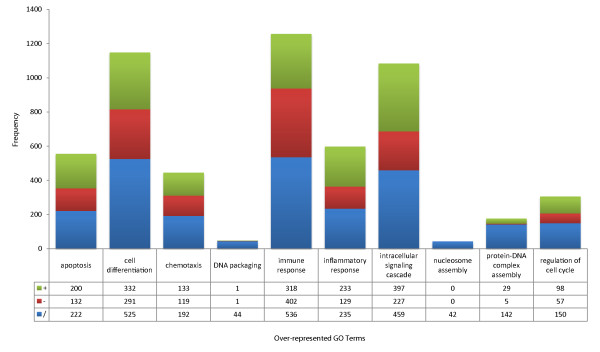
**Directionality of HIV-host interactions by functional category**. The frequency of interaction types classified as positive (green), negative (red) or neutral (blue) according to functional categories over-represented for HIV interactions; see Results for further details.

The HHPID data include the specific type of interaction between HIV and host proteins, for instance up- or down-regulation[3,4]. Combining this information with GO permits an analysis of how the various HIV genes perturb multiple host processes. We classified the 68 interaction types from HHPID into three polar categories: positive (denoted by +) for 'activated by', 'activates', 'enhanced by', 'enhances', 'stabilizes', 'stimulated by', 'stimulates', 'upregulated by' and 'upregulates'; negative (denoted by -) for 'cleavage induced by', 'cleaved by', 'cleaves', 'competes with', 'degraded by', 'degrades', 'disrupts', 'downregulated by', 'downregulates', 'inactivates', 'induces cleavage of', 'inhibited by' and 'inhibits'; with the remaining 25 interaction types as neutral (denoted by /)[2,4]. For most of the over-represented biological processes GO terms, we find the majority of interactions are mainly 'positive' in nature. The only exception, not surprisingly, is the immune response for which the majority of interactions are more 'negative' in nature (Figure 9). This demonstrates that HIV perturbs multiple cellular processes in multiple ways, that is, HIV appears to be both up- and down-regulating a wide range of proteins and functions.

## Discussion

Our results confirm that HIV preferentially interacts with hubs and bottlenecks - key host proteins that are apparently important to the cell (Figures 2 and 4). As proteins with a high connectivity and high betweenness centrality have previously been shown to demonstrate a tendency towards being essential [9-11,14-16] (and see Figures 4A and 4B), we investigated whether selection for interactions with essential proteins could account for these network topological observations. This was done by integrating phenotypic data - assessed with protein essentiality inferred from mouse knockout data - into our analysis. After correcting for ascertainment bias, however, we found no significant relationship between HIV-1 interaction and protein essentiality (Figure 5B). That is, HIV-1 proteins appear to be no more or less likely to interact with essential proteins than expected by random chance. This lack of over-representation of interactions with essential proteins (despite a significant tendency to interact with key host proteins) could be the result of ancestral selection pressure on retroviruses to minimise interactions with phenotypically essential proteins. Specifically, this would be consistent with selection acting on HIV's retroviral ancestors (due to longstanding co-evolution of retroviruses with host species) to minimise the pathogenic outcome of infection and maximise transmission potential, presumably in a trade-off between virulence and transmissibility [24,25].

Using an alternate measure of phenotype associated with perturbation: disease association, we investigated these observations further. Disease genes have previously been shown to display no propensity towards encoding either lowly or highly connected proteins [22] and we find that this is also true of the human protein interaction network when the overlap with essential genes is removed (Figure 6A and 6B). Accordingly, we would expect to observe no relationship between disease-association and HIV interaction amongst human proteins. Initially we find an over-representation of disease-association amongst HIV-interacting human proteins (Figure 7B). However, after compensating for ascertainment bias in the literature, we find the opposite: there appears to be an under-representation of disease-association amongst HIV-interacting proteins (Figure 7B). As there is no apparent relationship between connectivity and disease-association (Figure 6A), the under-representation of disease-association amongst HIV-interacting proteins is not related to network topology. Rather, we hypothesise that this under-representation of disease-association could again represent a selection pressure on retroviral proteins to avoid interacting with proteins associated with adverse phenotypes.

Given these results, how can we explain HIV's tendency to interact with high-degree and high-betweenness host proteins? Dyer and co-workers [8] have suggested that viral and bacterial proteins tend to interact with key proteins, as they may control critical human cellular processes, through their high connectivity and betweenness centrality. We find that the two concepts are interrelated: certain human proteins are central because they represent essential cellular functions, e.g., immune response. HIV interacts with these proteins to achieve its biology, and their high connectivity is simply secondary to this. Indeed, proteins involved in the over-represented biological process GO terms tend to be highly connected and central (Figure 8). Thus, HIV's propensity to interact with highly connected and central proteins is mainly a consequence of its interactions with particular cellular functions, rather than being related to global network properties in any straightforward way.

The specificity of the HIV-1 host interaction from HHPID, in the context of these underlying host protein functions, permits a detailed analysis of HIV's perturbation of the host system. Indeed, focussing on biological functions (from GO), our analysis demonstrates the directionality and complexity of both pro-pathogen (the majority promoting HIV's replication cycle) and pro-host (the host response to infection) interactions with specific cellular functions [3]. Collectively this highlights the subtle but complex manipulation of the host cell.

Throughout our analyses, we have corrected for the potential effect of ascertainment bias [20]. However, as it is very difficult to provide an accurate estimate for the degree of bias in the HHPID data, we have deliberately chosen a very conservative methodology for bias correction. Therefore, whilst we can be confident that degree and betweenness are both higher than expected after correction, it is possible that we are over-correcting in the case of the essentiality and disease-association data. Our results should therefore be interpreted as indicating no evidence for over-representation of these properties amongst HIV-interacting proteins; further research into bias correction methods for genome-scale data will be needed in order to provide more definitive conclusions.

## Conclusion

In order to fully understand HIV's hijack of the host system it will be necessary to study in detail the functional modules that are being exploited. This is exemplified by the complexity of HIV-host interactions, with the same functions being targeted multiple times (Figure 9). It will also be important to study the directionality of interactions, i.e., those that are pro-pathogen interactions as opposed to pro-host interactions[3], or even bystander interactions, incidental interactions of little consequence to either virus or host. Our finding that that there are patterns in terms of the types of interactions HIV makes can be explained by the cellular functions that HIV requires in order to replicate. The apparent tendency for HIV to 'avoid' phenotypically important molecules, underlines - despite HIV's recent acquisition by humans - the long-standing relationship that retroviruses have with their hosts. As more data become available, it will be informative to study this co-evolution of pathogens with their (often changing) host species. Understanding the precise molecular specificity of both the adaptation and persistence of pathogens with their hosts will yield novel insights into virulence and, potentially, new intervention strategies.

## Methods

### Main Data sets

Human protein interactions were derived from multiple sources: BioGRID http://www.thebiogrid.org, BIND http://www.bind.ca and HPRD http://www.hprd.org and filtered from the NCBI "interactions" file ftp://ftp.ncbi.nlm.nih.gov/gene/GeneRIF. Interaction data contained in these data sets are derived from multiple sources. HIV-host interactions and properties were derived from The HIV-1, Human Protein Interaction Database (available at http://www.ncbi.nlm.nih.gov/RefSeq/HIVInteractions). The data set currently comprises 1,435 human genes encoding 1,448 proteins that interact with 19 HIV-1 proteins making 2,589 unique interactions, curated from over 3,200 papers published between 1984 and 2007[2,4]. This paper also made extensive used of the "gene_info" and "gene2refseq" files provided by the Entrez Gene database ftp://ftp.ncbi.nlm.nih.gov/gene filtered to human genes (n = 36,455) and limited to those known to be protein-coding (n = 21,504). All data sets were current as of July 2009.

### Protein Essentiality

To predict the essentiality of a human gene, we used the phenotype information of the corresponding mouse ortholog. A human gene was defined as essential if a knockout of its mouse ortholog confers lethality. We obtained the human-mouse orthology and mouse phenotype data from Mouse Genome Informatics http://www.informatics.jax.org/[17]. We considered the annotations of postnatal, prenatal and perinatal lethality as lethal phenotypes, and the rest of the phenotypes as nonlethal ones. Overall, 27,697 annotations were filtered to leave 2,145 genes with an inferred essentiality.

### Disease Association

The Online Mendelian Inheritance in Man (OMIM) Morbid Map http://www.ncbi.nlm.nih.gov/sites/entrez?db=omim contains the most complete curated disorder-gene associations [18]. The data was filtered for the "(3)" tag http://www.ncbi.nlm.nih.gov/Omim/omimfaq.html#gene_map_symbols, for which there is strong evidence that at least one mutation in the particular gene is causative to the disorder, to identify 3,328 unique diseases across 3,049 genes. We used the gene_info file (to convert OMIM gene symbols to NCBI GeneIDs to facilitate integration. This data was used as a proxy for mild phenotypic effect.

### Gene Ontology

GO terms [23] were collected for each human gene from the NCBI "gene2go" file ftp://ftp.ncbi.nlm.nih.gov/gene/DATA. Term ancestors were then determined for each term from "gene_ontology_edit.obo" http://www.geneontology.org to ensure complete coverage. Select GO terms were taken from[3,23], retested for over-representation amongst HIV-interacting human proteins using Fisher's Exact Test in R[26] and separated into the three ontologies: biological process, cellular component and molecular function.

### Network Visualisations

Networks were visualised as graph-based layouts using Cytoscape [27].

### Degree, Hubs, Betweenness and Bottlenecks

The degree of a vertex in a network is the number of connections it has, in the case of a PPI network, this represents the number of other proteins the vertex interacts with. The degree of a single vertex is therefore equal to the number of adjacent edges.

A protein with a high degree is considered a hub and these have frequently been identified as the most vulnerable points in biological networks [9-11,14-16]. Yu et al. [16] classify a protein as a hub if it falls within the top 20% of proteins when sorted according to their degree. A cut-off of 20% in our data categorises a hub as any protein with a degree ≥3, we therefore chose a stricter cut-off of 2% so a hub is only classified as such with a degree ≥23.

Betweenness is a centrality measure of a vertex within a graph that summarises its relative importance both locally and globally [14-16]. Vertices that occur on many shortest paths between other vertices have higher betweenness than those that do not and are considered bottlenecks. Bottlenecks are generally a more accurate indicator of essentiality than degree or hub propensity[16], despite the two being correlated. For a graph *G *= (*V, E*), the betweenness centrality *C*_*B *_(*v*) for vertex *v *is: , where *σ*_*st *_is the number of geodesic (shortest) paths from *S *to *t*, and *σ*_*st *_(*v*) is the number of geodesic paths from *S *to *t*, that pass through a vertex *v*. We use Brandes' algorithm[12] to calculate the betweenness centrality of all vertices in *G*, normalised by dividing through the number of pairs of vertices not including *v*: (*V*-1)(*V*-2). As for hubs, we define a bottleneck as the top 2% of ranked proteins, so a bottleneck is classified as such with a normalised betweenness centrality ≥2.43 × 10^-4^.

The Wilcoxon rank-sum test, implemented in R [26], was used to compare the distributions of degree/betweenness across the entire genome against individual over-represented biological processes GO terms (see above). This enables us to determine whether the distributions for each GO term are significantly different to that found in the genome.

### Ascertainment bias

For every protein coding gene contained in Entrez Gene (n = 21,504), we obtained the number of unique publications using "Entrez Programming Utilities" http://eutils.ncbi.nlm.nih.gov/entrez/query/static/eutils_help.html. In total, 409,964 publications were recorded; with an average 19 articles per gene (2,217 genes were not matched to a publication).

Rejection sampling was used to generate sets of random genes that matched the publication frequency distribution of the HIV-interacting human set *f(x*) (n = 1,431), from the overall protein-coding gene population with publication frequency distribution *g*(*x*) (n = 21,504).

The John von Neumann Monte Carlo algorithm [28] was used, such that instead of sampling directly from the distribution *f(x*), we use an envelope distribution *Mg(x*), where *M *is the maximal *f(x) < Mg(x)*, and selected such that *f(x) < Mg(x) *for all observed publication counts *x*:

A) A gene (with publication count *x*) is selected at random from the overall population with publication frequency distribution *g*(*x*). A random number *U *from *U*(0,1) is also selected.

B) If , *x *is accepted as a realisation of *f(x) *and the gene is kept, otherwise sample step (A) is repeated.

The procedure is repeated until a set of genes of the required size is obtained. The samples match the distribution with a p-value of 0.43 (chi-squared, Figure 3). Using this procedure we constructed 10,000 sets of 1,431 randomised genes, *rand*_*(lit)*_, matching the publication frequency distribution of the HIV-interacting human genes. For comparison, 10,000 fully randomised samples, *rand*_*(pop)*_, were also generated by standard random sampling from the set of all genes. When comparing observed properties to these random samples, a z-score calculation was used to standardise the raw score *s *of each property tested,  and this was converted to a P-value using R [26]. This enables us to determine whether any results in the HIV-interacting set are due to ascertainment bias.

### Gene set enrichment analysis

Following the example of Dyer et al. [8], we adapted the gene set enrichment analysis (GSEA) method of Subramanian et al. [19] to test for significant differences between HIV-interacting and random sets of genes (both *rand*_*(lit) *_and *rand*_*(pop)*_). For a graph *G *= (*V*, *E*) let *L *be the list *V *ranked by either degree or by betweenness centrality. Let *S *be a subset of vertices within *L*, for example, the vertices that are HIV-interacting, *rand*_*(lit) *_or (*rand*_*(pop)*_. Let *l*_*i *_be the value (of degree or centrality) at index *i *of *L*, such that 1 ≤ *i *≤ |*L*|. If *i *is a member of *S*, the protein whose rank is *i*, thus, belongs to *S*. First, calculate , the sum of all the values of *S*. Next, for each index *i *of *L*, we compute two values, , the weighted fraction of proteins in *S *with an index ≤ *i *and , the fraction of proteins not in *S *with an index ≤ *i*. The enrichment score is therefore the largest positive value of *es(S, L) *= *P*_*hit *_(*S, i*) - *P*_*miss*_*(S, i)*. A large positive value of *es(S, L*) indicates that the proteins in *S *have high degree or high betweenness centrality. To compute p-values for the observed *es(S, L*), Dyer and co-workers [8] selected |*S*| random proteins from *L *1,000,000 times and estimated the p-value based on this distribution. However, we predict *S *to be biased, so similarly biased random samples |*S*| must be taken from *L*. We therefore used rejection sampling to generate 10,000 samples of |*S*_*HIV*_| with the distribution of *S*_*HIV *_in preference to the naïve random selection. A p-value was calculated from the z-score using R[26].

## Authors' contributions

All authors participated in the design of the study. JED carried out all analysis and drafted the manuscript. JWP helped with the statistical analysis. DLR conceived of the study. All authors edited and approved the final manuscript.
